# Ultra‐high static magnetic fields cause immunosuppression through disrupting B‐cell peripheral differentiation and negatively regulating BCR signaling

**DOI:** 10.1002/mco2.379

**Published:** 2023-10-01

**Authors:** Heng Gu, Yufan Fu, Biao Yu, Li Luo, Danqing Kang, Miaomiao Xie, Yukai Jing, Qiuyue Chen, Xin Zhang, Juan Lai, Fei Guan, Huamei Forsman, Junming Shi, Lu Yang, Jiahui Lei, Xingrong Du, Xin Zhang, Chaohong Liu

**Affiliations:** ^1^ Department of Pathogen Biology School of Basic Medicine Tongji Medical College and State Key Laboratory for Diagnosis and Treatment of Severe Zoonostic Infectious Disease Huazhong University of Science and Technology Wuhan China; ^2^ High Magnetic Field Laboratory Hefei Institutes of Physical Science Chinese Academy of Sciences Hefei Anhui China; ^3^ GeneMind Biosciences Company Limited Shenzhen China; ^4^ Department of Rheumatology and Inflammation Research Institute of Medicine Sahlgrenska Academy University of Gothenburg Goteborg Sweden; ^5^ Shanghai Key Laboratory of Metabolic Remodeling and Health Institute of Metabolism and Integrative Biology Fudan University Shanghai China; ^6^ Institutes of Physical Science and Information Technology Anhui University Hefei Anhui China

## Abstract

To increase the imaging resolution and detection capability, the field strength of static magnetic fields (SMFs) in magnetic resonance imaging (MRI) has significantly increased in the past few decades. However, research on the side effects of high magnetic field is still very inadequate and the effects of SMF above 1 T (Tesla) on B cells have never been reported. Here, we show that 33.0 T ultra‐high SMF exposure causes immunosuppression and disrupts B cell differentiation and signaling. 33.0 T SMF treatment resulted in disturbance of B cell peripheral differentiation and antibody secretion and reduced the expression of IgM on B cell membrane, and these might be intensity dependent. In addition, mice exposed to 33.0 T SMF showed inhibition on early activation of B cells, including B cell spreading, B cell receptor clustering and signalosome recruitment, and depression of both positive and negative molecules in the proximal BCR signaling, as well as impaired actin reorganization. Sequencing and gene enrichment analysis showed that SMF stimulation also affects splenic B cells' transcriptome and metabolic pathways. Therefore, in the clinical application of MRI, we should consider the influence of SMF on the immune system and choose the optimal intensity for treatment.

## Graphical abstract

33.0 T SMF treatment resulted in disturbance of B cell peripheral differentiation and antibody secretion, but lower magnetic fields, including 28.7, 17.8, or the 11.2 T SMFs had much less or no such effects. 33.0 T SMF treatment induced downregulate BCR proximal signaling and impaired F‐actin remodeling related BCR clustering. SMF stimulation affected the transcriptome and metabolic pathways of B cells.

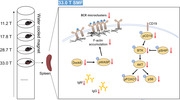

## 1 INTRODUCTION

The static magnetic field (SMF) is generated by steady currents or permanent magnets with constant magnetic field intensity and distribution. The intensity of magnetic fields varies in different situations, such as the earth's magnetic field is about 0.5 G (Gauss), which is 50 μT (Microtesla). In the last few decades, SMF has been widely applied in medicine, such as magnetic resonance imaging (MRI), which is one of the most common clinical diagnostic equipment due to its powerful imaging capability. Higher magnetic field intensity of MRI has been pursued to increase the resolution and sensitivity while reducing exposure time.^1^ In most hospitals, MRI strength ranges from 1.5 to 3.0 T, which has been proven to be nonhazardous.^2^ The strongest human MRI scanner of 10.5 T has been tested, and a 21.1 T small‐bore MRI has also been used on rodents.^3^, ^4^ The biosafety of SMF is crucial to its application. Previous studies have shown that SMF has a certain influence on animal reproduction,^5^, ^6^ behavior,^7^, ^8^ nerve,^9^ and immune system.^10^ And these effects are related to magnetic field intensity, direction, and gradient as well as sample exposure time.^11^ In biological effect studies, SMFs can be classified as weak, moderate, high, and ultra‐high in accordance with their magnetic intensity, which are less than 1 mT, 1 mT to 1 T, 1−20 T, and above 20 T, respectively.^11^ To date, the SMF biosafety studies have mainly focused on magnetic fields below 20 T, while studies above 20 T are very limited. Tian et al.^12^ examined the blood biochemistry, complete blood counts, key organ weights, and histomorphology of mice exposed to 7.0–33.0 T and found most parameters in the normal range. However, more thorough studies are still needed to investigate the biological effects of ultra‐high magnetic fields. In this study, we aimed to investigate the effects of exposure to ultra‐high SMF, especially with intensity ≥30 T in healthy adult mice. Four different magnetic field intensity stimulations were selected, 33.0 and 28.7 T, belonging to the ultra‐high SMF, while 17.8 and 11.2 T as controls, belonging to the high SMF group.

The immune system plays an important role in host defense as well as tissue homeostasis,^13^ and SMF has been shown to affect the immune system. SMF has potential antineoplastic^14^ and antiphlogistic influences,^15^ and SMFs with different parameters have various effects on the weight of immune organs and the total count of white blood cells in mice.^12^, ^16^, ^17^ As for innate immune cells, 0.6 T magnetic field^18^ can promote M2 polarization, probably because magnetism regulates the cytoskeleton by affecting the cell membrane and calcium channel receptor.^10^, ^19^ 10 T SMF exposure can reduce pro‐inflammatory cytokines secreting, such as IL‐6, IL‐8, and TNF‐α, and increase the secretion of anti‐inflammatory factors IL‐10 to some extent.^20^ In addition, a previous study reported that 0.4 T SMF increased NK cell cytotoxicity by activating multiple mitogen‐activated protein kinase pathways.^21^ As for adaptive immune cells, magnetic exposure remarkably increased the apoptosis of phytohemagglutinin‐stimulated lymphocytes.^20^ This might be due to the genotoxicity of SMF, its effects on microtubule assembly and energy metabolism disrupting cell division.^14^, ^20^, ^22^ The susceptibilities of naïve and memory CD4^+^ or CD8^+^ T cells to SMF exposure were different.^20^ 0.5 mT SMF exposure decreased IFN‐γ secretion, cell proliferation, and intracellular free calcium concentration in memory CD4^+^ T cells.^23^ 0.3 and 0.6 T SMFs enhanced the production of Granzyme B, IFN‐γ and TNF‐α in CD8^+^ T cells while promoting mitochondrial respiration to increase antitumor function.^24^ Although studies have recognized the importance of the influence of SMF exposure on the immune system, research has yet to systematically explore its effect on B lymphocytes.

B lymphocytes are essential for humoral immunity and play a crucial role in antibody secretion, antigen presentation, and immune regulation. The activation of B cell receptor (BCR) is essential for B cell function. The binding of specific antigens to the BCRs induces their aggregation by cross‐linking BCRs, then the conformation of BCR changes to expose the activation site, which triggers B cell activation and initiates an array of intracellular signal cascades.^25^ In the proximal signaling, the phosphorylated immunoreceptor tyrosine‐based activation motifs in Igα/β recruit and activate spleen tyrosine kinase and further recruit Bruton's tyrosine kinase (Btk), phospholipase C‐γ, and other downstream molecules with the help of lipid phosphatidylinositol 3,4,5‐trisphosphate.^26^ In addition, Vav activates the Wiskott‐Aldrich Syndrome protein (WASp, an actin nucleation‐promoting factor) to induce actin aggregation, and the cytoskeleton reorganization regulates BCR aggregation and provides a feedback regulation on the BCR signaling.^27^ The normal function of B cells is crucial to the immune system. It remains unclear whether SMF exposure affects BCR signaling transduction and actin reorganization.

In this study, we explored the effects of SMF on B cell differentiation by exposing mice to SMFs with four different intensities. Through flow cytometry assay, we found that ultra‐high SMF altered the differentiation of peripheral B cells and decreased the expression of IgM on B cell surface compared with high SMF, while no influence on the proliferation and apoptosis of B cells was observed. Meanwhile, we noticed that the proximal BCR positive and negative molecules, including CD19, Btk, and SHIP, were decreased after antigen stimulation in the ultra‐high SMF exposure group. With total internal reflection fluorescence microscopy (TIRFm), we found decreased BCR aggregation, impaired B cell expansion, and abnormal actin reorganization after antigen stimulation in the mice of ultra‐high SMF exposure group. Therefore, our study suggests that ultra‐high SMFs could disrupt B cell peripheral differentiation as well as negatively regulate BCR signaling and thereby cause immunosuppression.

## 2 RESULTS

### 2.1 Ultra‐high SMF interferes with the differentiation of peripheral B cells and inhibits antibody secretion

**FIGURE 1:**
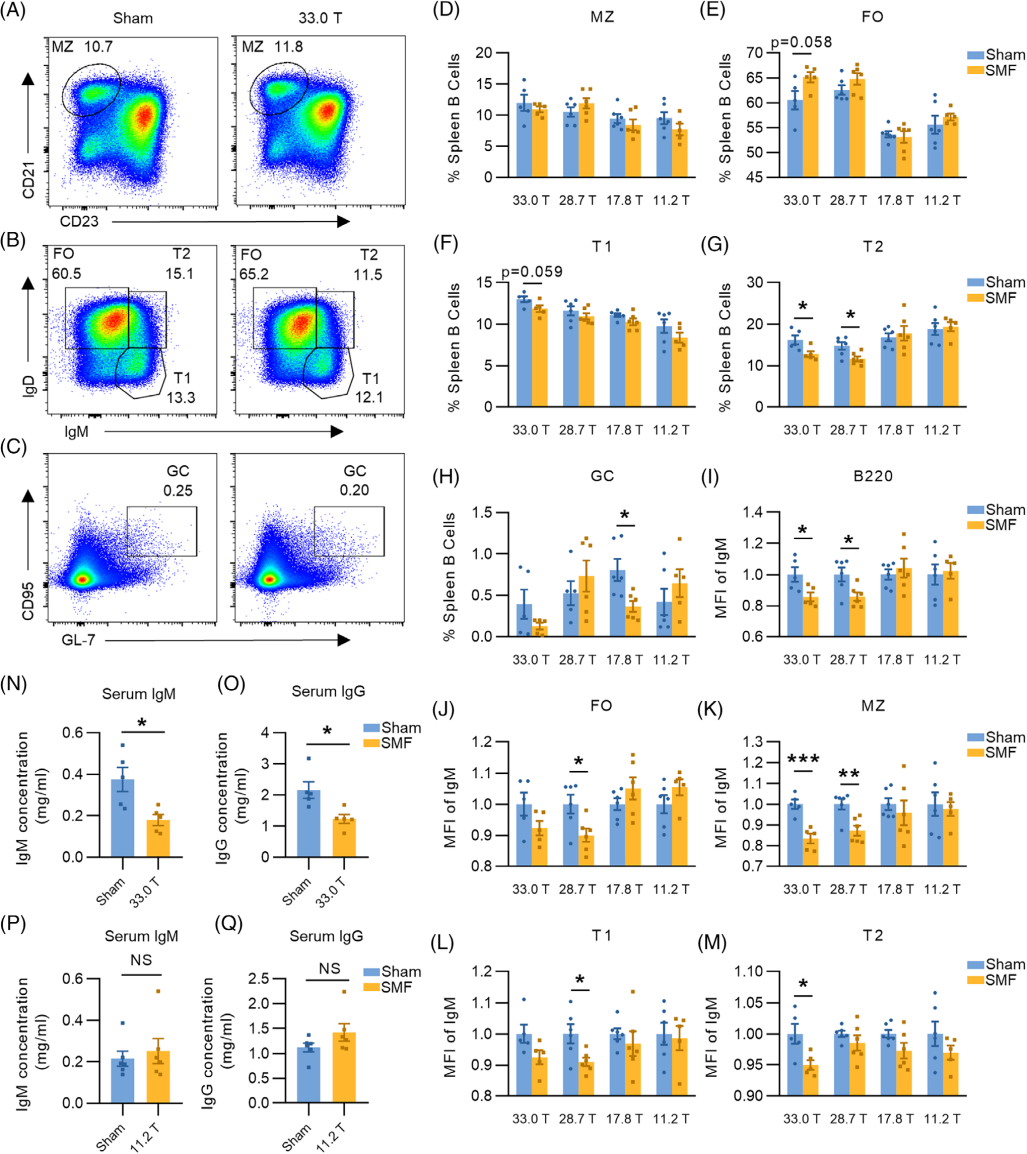
33.0 T ultra‐high static magnetic field interferes with the differentiation of peripheral B cells and inhibits antibody secretion. (A) Representative dot plots of peripheral marginal zone (MZ) B‐cell in splenic B cells from sham and 33.0 T SMF‐exposed groups. (B) Representative dot plots of peripheral B‐cell subsets including follicular (FO), transitional type‐1 (T1), and transitional type‐2 (T2) B cell in splenic B cells from sham and 33.0 T SMF‐exposed groups. (C) Representative dot plots of peripheral germinal center (GC) B‐cell in splenic B cells from sham and 33.0 T SMF‐exposed groups. (D–H) Shown are average percentages of MZ, FO, T1, T2, and GC B cells from sham and 33.0 T/28.7 T/17.8 T/11.2 T SMF‐exposed groups. (I) IgM expression in splenic B cells from sham and 33.0 T/28.7 T/17.8 T/11.2 T SMF‐exposed groups. (J–M) Mean fluorescence intensity (MFI) of IgM in FO, MZ, T1, and T2 B cells from sham and 33.0 T/28.7 T/17.8 T/11.2 T SMF‐exposed groups. (N and O) The concentration of IgM/IgG in serum of mice in sham and 33.0 T SMF‐exposed groups (*N* = 5). (P and Q) The concentration of IgM/IgG in serum of mice in sham and 11.2T SMF‐exposed groups (*N* = 6). Sample were analyzed with FlowJo software, Error bars were shown as mean ± SEM. Dots represent individual mouse. *p < 0.05; **p < 0.01; ***p < 0.001 and ns: no statistical significance.

We first examined the differentiation of peripheral B cells through flow cytometry under 33.0, 28.7, 17.8, and 11.2 T magnetic field exposure and found that compared with the sham group, the percentage of marginal zone (MZ) B cells had no significant changes (Figures 1A and D). However, we found a slight effect on the development of follicular (FO) and transitional type‐1 (T1) B cells at 33.0 T, which was not detected at other intensities (Figures 1B, E, and F). The percentage of T2 subgroups was significantly lower in both 33.0 T and 28.7 T ultra‐high SMF groups than their paired sham groups, while no differences were found at the other two high magnetic fields (Figures 1B and G). In addition, we also found a reduced percentage of the germinal center (GC) subset in the 17.8 T group (Figure 1C and H). Interestingly, we also found that 33.0 and 28.7 T magnetic fields exposure could reduce the IgM expression in splenic B cells, but 17.8 and 11.2 T SMF exposure showed no obvious effect (Figure 1I). Therefore, we examined the mean fluorescence intensity (MFI) of IgM in subsets of peripheral B cells, which indicated that 33.0 T SMF exposure reduced IgM expression in MZ and T2 B cells and 28.7 T SMF exposure reduced that in FO, MZ, and T1 B cells. Consistent with total splenic B cells, no clear differences were observed in IgM expression within each subset in 17.8 and 11.2 T SMF compared with the sham groups (Figures 1J–M). These results suggested that ultra‐high SMF exposure might have a potential inhibitory effect on B cell differentiation.

We also measured the antibody content in serum to explore the effect of SMF on antibody secretion of B cells. IgM is the first antibody produced in the initial humoral immune response and is the vanguard of the infection resistance in the organism. Results showed that the concentration of IgM in the serum of mice in 33.0 T group was significantly lower than that in the sham group (Figure 1N). And IgG is the main antibody produced in the secondary humoral immune response, which is broadly distributed in the body and plays an important immune role. Similarly, 33.0 T exposure caused a decrease in IgG concentration in serum (Figure 1O). However, there were no significant changes in the concentration of either IgM or IgG after 11.2 T SMF exposure (Figures 1P and Q), as well as after 28.7 and 17.8 T exposure (Figures S2A–D), suggesting that the antibody secretion may be influenced by the intensity of the magnetic field.

**FIGURE 2:**
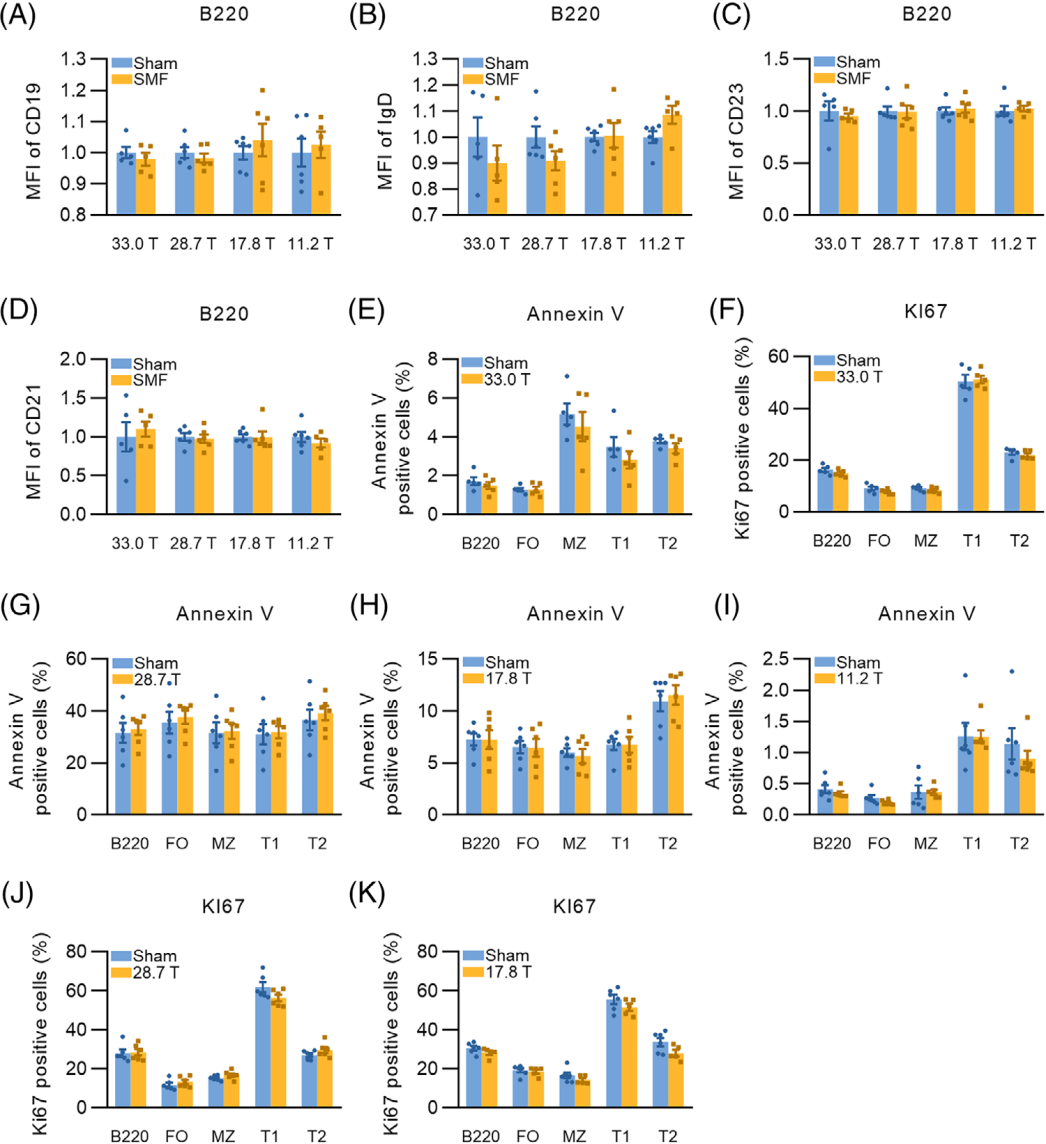
SMF exposure has little effect on peripheral B cell proliferation, apoptosis and the expression of CD19, IgD, CD23, and CD21 on B cells. (A–D) MFI of CD19, IgD, CD23, and CD21 in splenic B cells from sham and 33.0 T/28.7 T/17.8 T/11.2 T SMF‐exposed groups. (E and F) Percentage of Annexin V and Ki67‐positive cells in total splenic B cell and FO, MZ, T1, T2, B subsets in sham and 33.0 T SMF‐exposed groups. (G–I) Percentage of Annexin V positive cells in total splenic B cell and FO, MZ, T1, T2, B subsets in sham and 28.7 T/17.8 T/11.2 T SMF‐exposed groups. (J and K) Percentage of Ki67‐positive cells in total splenic B cell and FO, MZ, T1, T2, B subsets in sham and 28.7 T/17.8 T SMF‐exposed groups. Samples were analyzed with FlowJo software, Error bars were shown as mean ± SEM. Dots represent individual mouse. *p < 0.05; **p < 0.01; ***p < 0.001 and ns: no statistical significance.

For other cell surface molecules (CD19, CD21, CD23, and IgD), most of them were normal either in total splenic B cells or in each subset under ultra‐high and high magnetic field exposures (Figures 2A–D and S1A–P). Annexin V and Ki67 represent cell apoptosis and proliferation, respectively, and we did not observe any effects of SMF exposure with 4 different intensities on Annexin V and Ki67 expression in total splenic B cell as well as in each subset (Figures 2E–K).

Overall, exposure to ultra‐high SMF interferes with the differentiation and development of peripheral B cells and has an impact on some important cell surface molecules, such as IgM, which might influence cellular function. Meanwhile, 33.0 T SMF exposure also decreases the concentration of IgM and IgG and thus might weaken the humoral immune response. But the impacts of SMFs with different intensities on apoptosis and proliferation have not been found yet. In addition, in the high intensity magnetic field exposure groups (17.8 and 11.2 T), no clear difference was found from the sham groups in the above aspects, which might indicate that the inhibition of SMF on the differentiation of peripheral B cells and the expression of important surface molecules may be SMF intensity dependent, with a greater effect at higher magnetic field intensity.

### 2.2 33.0 T SMF exposure suppresses proximal BCR signaling

**FIGURE 3:**
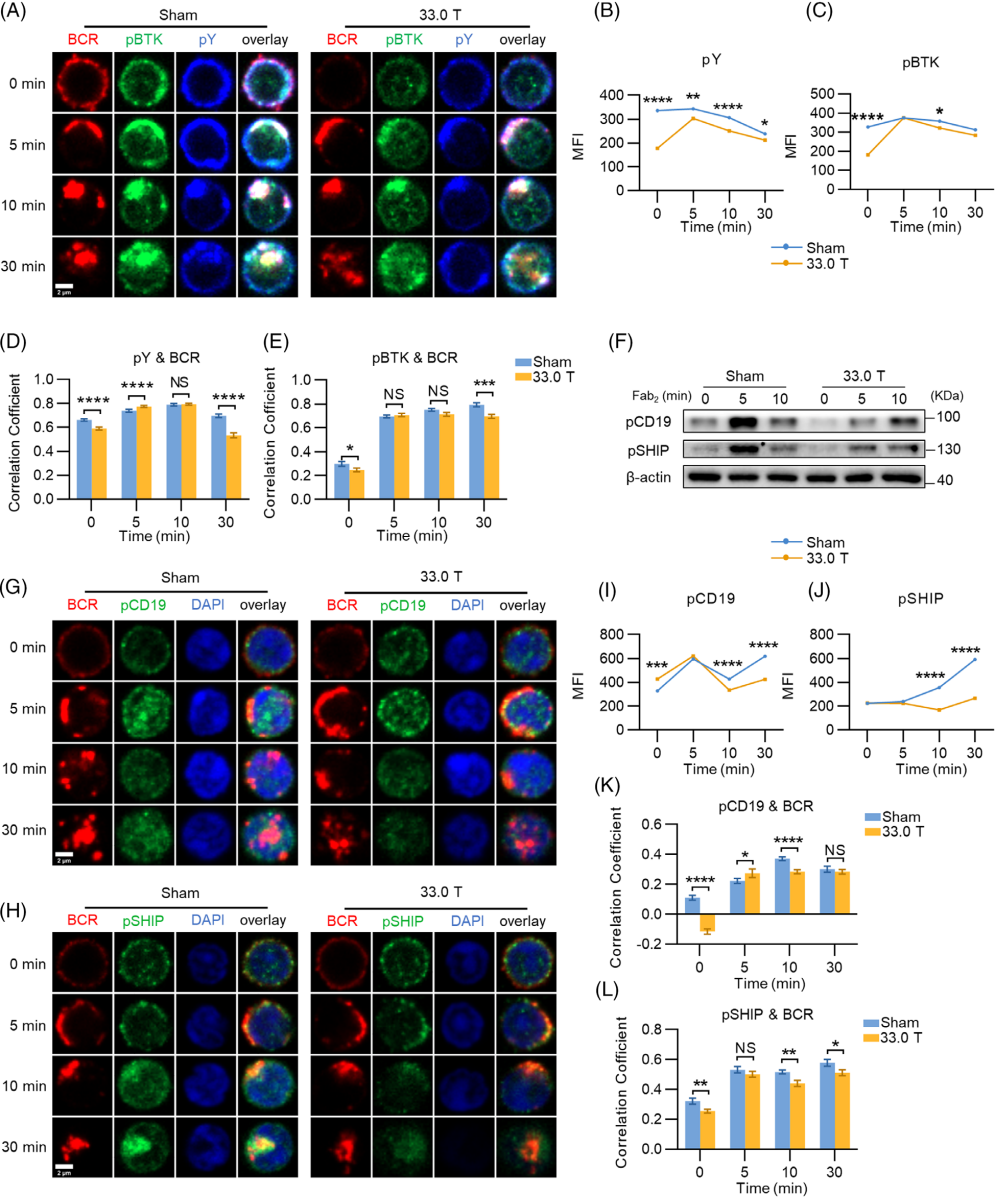
33.0 T ultra‐high static magnetic field exposure suppresses proximal BCR signaling. Splenic B cells from sham and 33.0 T SMF‐treated mice were stimulated with AF594 F(ab′)2 goat anti‐mouse IgG + IgM (10 μg/mL) for 0, 5, or 10 min, then fixed, permeabilized, and stained for markers following. (A) Representative confocal microscopy (CFm) images of phosphotyrosine (pY), phosphorylated BTK (pBTK) and BCR (60× objective, scale bar = 2 μm). (B and C) CFm analysis of MFIs of pY and pBTK. (D) Colocalization between pY and BCR. (E) Colocalization between pBTK and BCR. The colocalization was analyzed using Pearson's correlation coefficient. (F) Western blotting of phosphorylated CD19 (pCD19) and phosphorylated SHIP (pSHIP) levels in splenic B cells activated biotin‐conjugated F(ab’)2‐anti‐mouse‐Ig (M + G) and streptavidin for 0, 5, and 10 min. (G and H) Representative CFm images of pCD19 and pSHIP (60× objective, scale bar = 2 μm). (I and K) MFIs of pCD19 and colocalization between pCD19 and BCR. (J and L) MFIs of pSHIP and colocalization between pSHIP and BCR. *p < 0.05; **p < 0.01; ***p < 0.001; ****p < 0.0001 and ns: no statistical significance.

After B cells receive antigen stimulation, BCRs aggregate into clusters and recruit BCR signalosome associated molecules, which mediate the cascading BCR signaling and ultimately activate B cells. And BCR signaling plays a pivotal role in the differentiation and development of B cells. Thus, we used confocal microscopy (CFm) to continually observe the antigen internalization and presentation following BCR stimulation, as well as the participation and expression intensity of signaling molecules in the internalization process of B cell activation via colocalization analysis. The content of phosphotyrosine proteins (pY) could reflect the overall level of BCR signaling, and BTK is a crucial downstream molecule. Therefore, to investigate the impact of ultra‐high SMF on BCR signaling, we examined the spatiotemporal expression of pY and the activation of BTK in splenic B cells of mice in 33.0 T group and sham group with CFm at 0, 5, 10, and 30 min of soluble antigen (sAg) stimulation. We found noticeable decreases in the level of pBTK and pY in 33.0 T group after stimulation (Figures 3A, B, and C). Along with this, compared with the sham group, the colocalization between pY and BCR was significantly reduced at 0 and 30 min (Figures 3A and D), and the colocalization between pBTK and BCR was found to be reduced at 30 min of stimulation as well (Figure 3E). CD19 is a coreceptor of BCR, which has been known to be critical for B cell survival.^28^ Colocalization of pCD19 with BCR in exposure mice was significantly lower than in sham group at 10 min after sAg stimulation (Figures 3G and K). Similarly, we also detected the activation of a negative regulator of proximal BCR signaling SHIP‐1. We found a decreased colocalization of pSHIP‐1 and BCR in the exposed group at 10 to 30 min following stimulation (Figures 3H and L). Additionally, the levels of pCD19 and pSHIP were also decreased during 10 and 30 min (Figures 3I and J). Meanwhile, the expression levels of pCD19 and pSHIP were much lower at 5 min after sAg stimulation in 33.0 T exposure group than those in the sham group (Figure 3F). All these results suggest that ultra‐high SMF exposure negatively regulates the spatiotemporal expression of key signaling molecules to restrain proximal BCR signaling.

### 2.3 Decreased BCR clustering and signalosome recruitment in B cells from 33.0 T SMF‐exposed mice leads to the downregulation of BCR signaling

**FIGURE 4:**
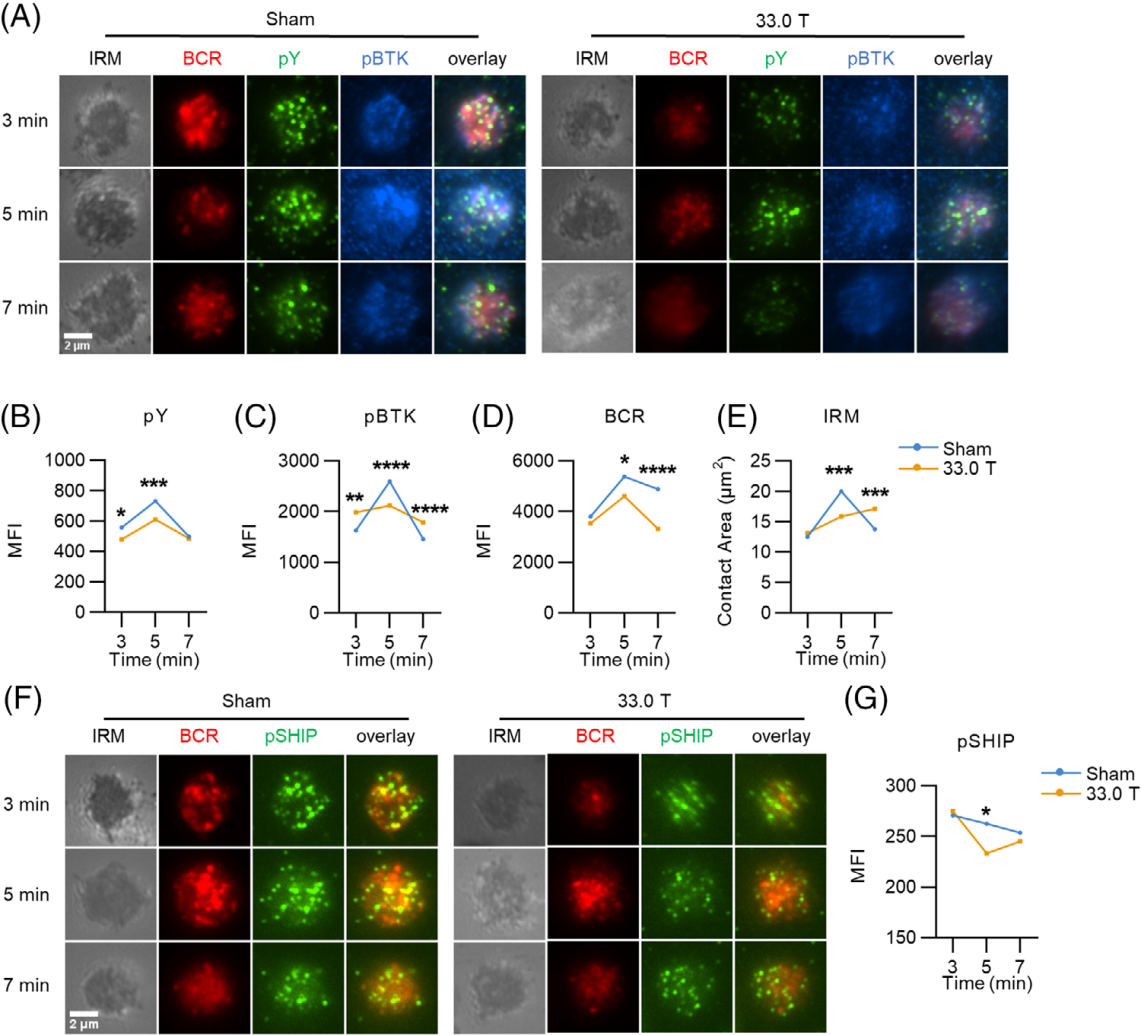
33.0 T ultra‐high static magnetic field exposure suppresses BCR clustering and decreases signalosome recruitment. (A) Representative total internal reflection fluorescence microscopy (TIRFm) images of pY and pBTK at 3, 5, or 7 min of activation with 10 μg/mL AF546 F(ab’)2 anti‐mouse Ig (M+ G) (100× objective, scale bar = 2 μm). (B and C) Quantitative analysis of pY and pBTK MFI. (D and E) MFI of BCR and B‐cell contact area were quantified using NIS‐Elements AR 5.0.1 software. (F) Representative TIRFm images of pSHIP (100× objective, scale bar = 2 μm). (G) Quantitative analysis of pSHIP MFI. *p < 0.05; **p < 0.01; ***p < 0.001; ****p < 0.0001 and ns: no statistical significance.

The early events of B cell activation, including BCR clustering and signalosome recruitment, are crucial for the conduction of BCR downstream signaling. To investigate the influence of ultra‐high SMF on the early activation of B cells, we used TIRFm to assay the formation of BCR clusters and other events close to the membrane in 33.0 T and sham groups when membrane antigen (mAg) stimulated for 3, 5, and 7 min. The area of the cell contact zone indicated the spreading of B cells. Additionally, it was significantly smaller in the SMF‐exposed group than in the sham group at 5 min (Figures 4A, E, and F). Moreover, the MFI of the BCR, which represented the aggregation of BCR, was reduced in 33.0 T exposure group at 5–7 min after stimulation (Figures 4A, D, and F). For the overall level of BCR signaling, the MFI of pY reached the peak at 5 min, and it was lower in the cells of exposed mice at 5 min after mAg stimulation (Figures 4A and B). We also found that the MFI values of pBtk and pSHIP in 33.0 T group were reduced compared with those of sham group at 5 min (Figures 4A, C, F, and G). All these results suggest that ultra‐high SMF stimulation weakens BCR signaling conduction by inhibiting BCR clustering and signalosome recruitment.

### 2.4 33.0 TSMF stimulation impedes the accumulation of F‐actin

**FIGURE 5:**
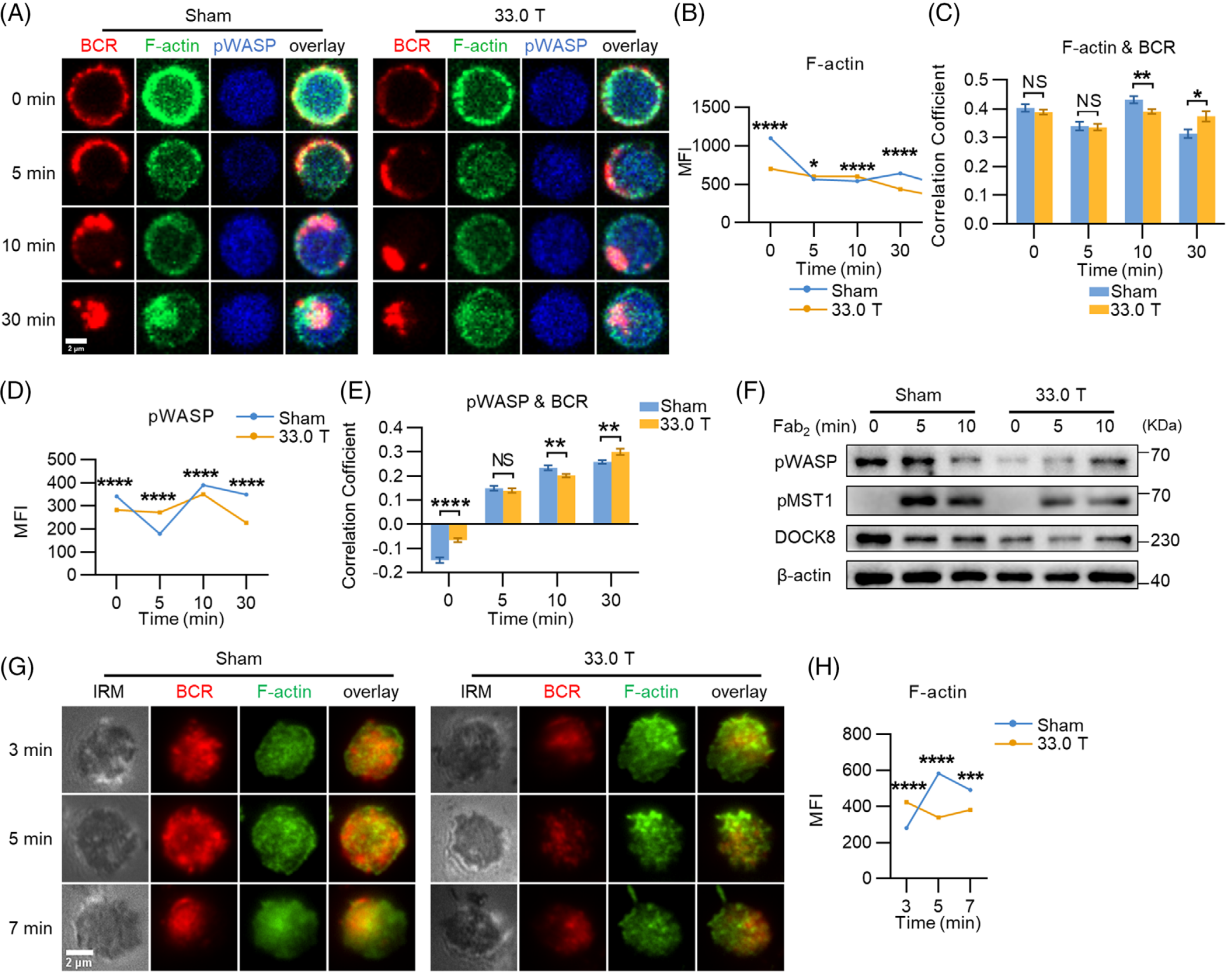
33.0 T ultra‐high static magnetic field stimulation impedes the accumulation of F‐actin. (A) Representative confocal microscopy (CFm) images of phosphorylated WASP (pWASP) and F‐actin (60× objective, scale bar = 2 μm). (B and C) MFIs of F‐actin and colocalization between F‐actin and BCR. (D and E) MFIs of pWASP and colocalization between pWASP and BCR. (F) Western blotting of pWASP, pMST1, and DOCK8 levels in splenic B cells with F(ab’)2 activation. (G) Representative total internal reflection fluorescence microscopy (TIRFm) images of F‐actin at 3, 5, or 7 min of activation with 10 μg/mL AF546 F(ab’)2 anti‐mouse Ig (M+ G) (100× objective, scale bar = 2 μm). (H) MFI of F‐actin in the contact zone. *p < 0.05; **p < 0.01; ***p < 0.001; ****p < 0.0001 and ns: no statistical significance.

Earlier studies have shown that the stimulation of SMF with low or moderate intensity affects the orientation, assembly and content of actin filaments.^29^, ^30^, ^31^ And the reorganization of actin has been proven to exert a crucial role in B cell activation.^32^ F‐actin is involved in receptor aggregation, signal molecule recruitment, and influencing BCR signaling positively or negatively. In addition to this, F‐actin also affects B cell adhesion and migration through controlling B cell morphology. Besides that, BCR signaling regulates F‐actin mediated by WASp.^27^Thus, to explore the effect of SMF with ultra‐high intensity on B cell actin cytoskeleton, we detected the levels of activated WASp and F‐actin as well as their colocalization with BCR via CFm at different time points after sAg stimulation. Colocalization between F‐actin and BCR was significantly lower in 33.0 T exposure group at 10 min after stimulation than that in the sham group (Figures 5A and C). Meanwhile, the level of F‐actin in B cells of 33.0 T group declined obviously after 30 min of stimulation (Figures 5A and B). Colocalization of pWASp and BCR was less in the 33.0 T group compared with that in the sham group at 10 min (Figures 5A and E), and the MFI of pWASp was also decreased markedly from 10 to 30 min after stimulation (Figures 5A and D). We conducted another assay using TIRFm with higher resolution. The data show that the content of F‐actin in the contact area of B cells in the 33.0 T group was reduced at 5–7 min after the treatment of B cells with mAg (Figures 5G and H). Dock8 regulates actin accumulation by means of WASp,^33^ and studies have proven that Mst1 and WASp regulate each other through indirect effects.^34^ Interestingly, we noticed that the expression of pWASp in 33.0 T group was decreased at 5 min compared with sham group (Figure 5F). The expressions of pMst1 and Dock8 in 33.0 T group were lower than that in sham group at 5 and 10 min after stimulation (Figure 5F). This indicates that 33.0 T SMF exposure has an inhibitory effect on Mst1–Dock8–WASp axis. Altogether, the above results suggest that ultra‐high SMF exposure decreases F‐actin accumulation and that the potential mechanism for this process may be due to the repression of the Mst1–Dock8–WASp axis in the downstream of BCR singling caused by SMF exposure.

### 2.5 Exposure to SMFs affect the transcriptome profile of B cells and disturbs metabolism‐related molecules

**FIGURE 6:**
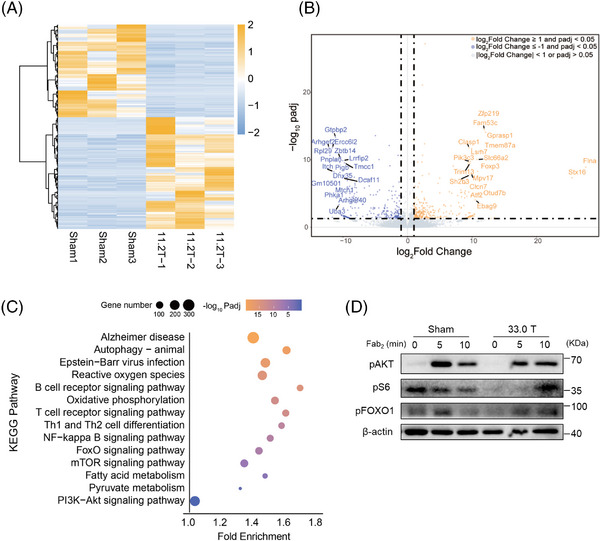
Static magnetic fields exposure impacts cell metabolism. (A) Heat map of differential transcripts between sham and 11.2 T SMF‐exposed groups. (B) Volcano plot of differentially expressed mRNAs, marking the top 100 differential genes with padj < 0.001. Blue dots represent downregulated genes (padj < 0.05 and |log 2 (FoldChange)| ≤ −1) and orange represents upregulated genes (padj < 0.05 and |log 2 (FoldChange)| ≥ 1). (C) Significantly differential transcripts enriched to 14 KEGG signaling pathways. (D) Western blotting of pS6, pAKT, and pFOXO1 levels in splenic B cells activated biotin‐conjugated F(ab’)2‐anti‐mouse‐Ig (M + G) and streptavidin for 0, 5, and 10 min in splenic B cells from sham and 33.0 T SMF‐exposed groups.

To investigate the possible mechanisms for the effects of SMF stimulation on B cell function, we performed transcriptome sequencing and KEGG analysis of B cells from 11.2 T SMF exposure group and sham group. Compared with the sham group mice, a total of 305 upregulated transcripts and 253 downregulated transcripts were detected in 11.2 T SMF‐treated mice B cells (Figure 6A). We tagged the top 100 differential genes with padj less than 0.001 (Figure 6B). To better understand the impact of SMF stimulation, we enriched the differentially expressed genes to 14 KEGG pathways, where *p* values indicate the degree of significant enrichment (Figure 6C). We found high enrichment in immune‐related pathways, such as B cell and T cell receptor signaling pathways and Th1 and Th2 cell differentiation, which strongly affected the regulation of humoral and cellular immunity. NF‐kappa B signaling pathway regulates genes involved in immunity, inflammation, and cell survival. Some other important intracellular signaling pathways were also highly enriched, especially FoxO, mTOR, and PI3K–Akt signaling pathways, and they perform essential regulatory roles in cell growth and metabolism, cytoskeleton, and other processes. Moreover, some fundamental cellular processes, such as autophagy, were also enriched. As for metabolic pathways, oxidative phosphorylation, fatty acid, and pyruvate metabolism were enriched to some extent. These results suggested that 11.2 T magnetic field exposure may affect the immune system in diverse ways, including cell differentiation, metabolism, and important signaling pathways. Earlier sequencing studies on human embryonic lung fibroblasts under 3.0 T magnetic field exposure found no obvious difference in protein‐gene expression between experimental and control groups,^35^ which also indicated that the effect of SMF might be intensity dependent. Since we sequenced transcriptome 2 months after the treatment of mice, this is an indication of a possible long‐term influence of high SMF on the transcriptional profile of B cells.

To further verify the effect of SMF on B cell metabolism, we detected the effect of 33.0 T ultra‐high SMF stimulation on the metabolic signaling pathway of splenic B cells. We lysed splenic B cells after sAg stimulation and analyzed the content of metabolism‐related molecules with western blotting. PI3K–Akt–mTORC1 is an essential metabolic signaling pathway in B cells, and Akt is a key regulator in it. The outcome showed that the expression of pAkt in 33.0 T group was significantly lower than the sham group at 5 min (Figure 6D). S6 is the downstream molecule of mTORC1 related to protein synthesis, and FOXO1 is a glucose metabolism‐related transcription factor in BCR distal signaling. The levels of pS6 and pFOXO1 in 33.0 T group were also decreased at 5 min (Figure 6D). Therefore, 33.0 T SMF exposure may affect B cell metabolism.

The above results indicate that both high and ultra‐high SMF stimulation could affect B cell metabolism to a certain degree, especially after 33.0 T stimulation, the metabolic signals were significantly reduced. SMF alters B cell homeostasis and makes B cell immunosuppressive by interfering with cellular metabolism, but this still needs to be further verified.

## 3 DISCUSSION

The field strength of SMFs in MRI has been enhanced significantly in the past few decades for better imaging resolution and detection capability. As a widely used technique in medicine, the effects of SMF on organisms has also been extensively studied, but its effects on B cells has not been explored. In this study, we first established the effects of magnetic fields on humoral immunity. Due to the technical limitations of magnetic field intensity, previous studies on the biological effects of SMF have mainly focused on low and moderate intensities. Earlier studies have found that SMF could affect natural immune cells, for instance, promoting M2 polarization of macrophages.^18^, ^19^ Furthermore, studies on the roles of SMF on adaptive immune cells concentrated on T cells and revealed that SMF could affect the number of stimulated CD4+ T cells and CD8+ T cells and the secretion of cytokines.^20^, ^23^, ^24^ It has also been known the implications of SMF on the balance between pro‐ and anti‐inflammatory molecules^20^ and its potential antitumor activity.^36^ A previous study has shown a significant reduction in spleen size in mice after 2 months of 33.0 T SMF exposure,^12^ which suggested that ultra‐high magnetic field might have a long‐term effect on immune organs, but its effects on humoral immunity have not been further explored. As a widely used technique in medicine, the effect of SMF on organisms has also been extensively studied, but its effect on B cells has not been explored. In this study, we first established the effects of magnetic fields on humoral immunity, filling the blank in the field of side effects of magnetic fields on B cells.

Meanwhile, our group has published several articles about long‐term treatment with magnetic fields and found that SMFs do not have many safety issues for healthy mice. For example, we exposed healthy mice continuously to 0.1–0.2 T SMF for 1.7 years and did not reveal harmful effects.^37^ We also found that high SMFs of 9.4 T exposure for 200 h did not have observable defects in tumor‐bearing mice.^38^ However, 14 h of prolonged treatment of gradient high SMFs had negative effects on type 1 and 2 diabetes mice, including spleen, hepatic, and renal tissue impairment.^39^ We have summarized the effect of long‐term SMF exposure in a book chapter recently, which can provide more information on this topic.^40^ The main purpose of this article is to investigate the effects of ultra‐high magnetic fields up to 33.0 T, hoping to provide information for the future development of high‐field MRI and other related devices, so our study was conducted at SMFs with four high or ultra‐high intensities, responding to the current demands for higher intensity magnetic fields in medical applications. Considering that the current clinical MRI detection time is about 0.5–1 h, 1‐h magnetic field treatment is used as the experimental condition here. And the sham groups were designed to exclude the influence of irrelevant factors, such as the environment and noise produced by the magnetic field‐generating equipment.

Our results revealed that ultra‐high SMF exposure could cause immunosuppression. 28.7 and 33.0 T magnetic resulted in abnormal B cells peripheral growth and differentiation, such as decreases in the proportion of T2 subset and mIgM expression. And 33.0 T exposure reduced the concentration of IgM and IgG in serum, which might cause a weakened humoral immune response. These effects were intensity dependent. We also explored the changes in BCR signaling and F‐actin in the splenic B cells of 33.0 T group after antigen stimulation for the detailed mechanisms. Exposure to ultra‐high magnetic field decreased BCR clustering and proximal positive and negative signalosome aggregation after stimulation, which suppressed the conduction of BCR signaling. Humoral immune response demands optimal BCR signaling, and both higher and lower BCR signaling may cause reduced response.^41^, ^42^ The α‐helix structure has been proved to give the protein diamagnetic anisotropy.^43^ In humans, the structure of the IgM–BCR complex was resolved by cryo‐EM, which revealed that the transmembrane region of mIgM and Igα/Igβ formed a four‐helix bundle that appeared to be conserved among all BCR isoforms.^44^ This may be related to the inhibition of BCR aggregation by ultra‐high intensity magnetic fields. Similar to previous studies, F‐actin accumulation was reduced after magnetic field exposure, as it can form organized polymers, which have strong antimagnetic anisotropy. Besides, the activation of Mst1–Dock8–WASp axis‐related molecules was depressed after antigen stimulation in 33.0 T group, probably leading to reduced accumulation of F‐actin. Thus, we speculate that ultra‐high SMF directly acts on the BCR complex and F‐actin to disrupt the activity of them, and that the interaction between BCR signaling and F‐actin aggregation may also be involved in this process. However, although more detailed mechanisms need to be further explored, our data here show that ultra‐high SMF causes immunosuppression by inhibiting BCR signaling and the peripheral differentiation of B cells.

Transcriptome analysis highlighted the alteration in BCR signaling and other important intracellular signaling pathways, further confirming our experiments of mice model. On the other hand, the enrichment of metabolic pathways suggested that SMF might affect B cell metabolism, as confirmed by the immunoblotting experiment we performed later, which found that SMF exposure inhibits the activation of PI3K–Akt axis‐related molecules. Magnetic fields have been shown to impact glucose and lipid metabolism in mice, such as downward moderate SMF treatment resulted in lower blood cholesterol and glucose levels and reduced body weight and lipid accumulation in the liver in type 2 diabetic mouse models,^45^ while 33.0 T SMF cause a 24.3% increase in cholesterol in exposure group compared with the sham group.^12^ Other studies have come to different or even opposite conclusions,^46^, ^47^ pointing to the fact that the effect of magnetic on metabolism was influenced by magnetic field parameters including intensity and direction. Moreover, SMF may also prevent the progression of high‐fat‐diet‐induced diabetes to some extent.^45^ Research over the past decade has demonstrated that B cells play a central role in chronic inflammation and immune dysregulation associated with obesity, while immune cell interaction and inflammation in the adipose tissue regulate systemic glucose and fatty acid metabolism.^48^ The PI3K–Akt signaling pathway promotes B cell anabolism and activation.^49^ However, it remains unclear whether impaired B cell metabolism affects adipose tissue and thus alters organism metabolism. Therefore, obesity models or type 2 diabetes models can be induced and further studied.

In conclusion, ultra‐high SMF exposure leads to immunosuppression, reduces serum concentration of IgG and IgM, interferes with peripheral B cell development and signaling, and has some inhibition of metabolic signaling pathways. More studies exploring the effects of different magnetic field parameters, including direction, intensity, gradient, and exposure, are expected. Although magnetic field is an effective medical treatment, attention should be paid to the side effects of ultra‐high SMF on the organism, and the best combination of magnetic field parameters should be selected.

However, the main limitation of this study is that we could not obtain data with a larger sample size. Due to the very high cost of electricity for performing these ultra‐high magnetic field experiments in the water‐cooled magnet, the small‐bore size of the ultra‐high magnet, as well as the very limited machine time available for these ultra‐high magnets, there are very few reports about >20 T ultra‐high magnetic field experiments on animals. It is more difficult to achieve large samples, especially for research on SMF above 30 T. Due to the failure of library construction, we did not obtain sufficient ultra‐high magnetic field sequencing data. We sequenced only one 28.7 T SMF‐treated mouse (data not shown). In order to verify the findings from 11.2 T SMF‐treated mouse, we measured mitochondrial function and the content of the key molecules in PI3K–Akt–mTORC1 pathway to further investigate the metabolic effects of ultra‐high magnetic field exposure. Although the results indicated that the metabolic signaling of B cells was impaired after high and ultra‐high magnetic field stimulation, the possibility that SMF stimulation may impact B cell function by impairing cellular metabolism still needs to be further verified. In addition, we seldom explore the influence of magnetic fields on the physiological functions of B cells, such as cytokine secretion and specific antibody production, which remain to be further investigated to help us better understand the effects of ultra‐high SMF exposure on B cells.

## 4 MATERIALS AND METHODS

### 4.1 SMF exposure system

The water‐cooled magnet (WM#5) of the National Major Scientific and Technological Infrastructure Construction Project in China, Chinese Academy of Sciences, Hefei, was used. The SMF exposure device consists of three parts. The inner part is a cylinder stacked with eight nonmagnetic stainless tubes, each containing one mouse. The middle is a precise temperature controlling and monitoring equipment with a double layer. And the outer is a water‐cooled magnet to provide SMF. Moreover, sham‐exposed groups were treated in the same environment as their corresponding SMF‐exposed groups. Detailed assembly and parameters have been described previously.^12^

### 4.2 Mice

Forty‐six healthy 8‐week‐old male C57BL/6 mice were placed on four different layers in the water‐cooled magnet randomly. The magnetic field intensity of each layer is 33.0, 28.7, 17.8, and 11.2 T respectively, with an upward magnetic field gradient. For the mice in each layer, they were divided into sham‐exposed group (*N* = 5 for 33.0 T sham group and *N* = 6 for the other groups) and SMF‐exposed group (*N* = 5 for 33.0 T group and *N* = 6 for the other groups). We exposed mice to the device for 70 min as described in the previous experiment.^12^ For each group, six mice in total were treated in six independent experiments over 3 days. Two months after the exposure, the spleens of mice were taken for isolating and analyzing B cells. During the experiment, the mice were housed in a standard barrier environment with a 12‐h light/dark cycle and ad libitum feeding.

### 4.3 ELISA

Serum was collected from the mice of SMF‐exposed groups and their corresponding sham‐exposed groups. The QuantiCyto® Mouse IgM ELISA kit (EMC 129; NeoBioscience) and Mouse IgG (Total) ELISA Kit (RK00375; abclonal) were used to measure the concentration of IgM and IgG in serum in accordance with the experimental protocol provided by manufacturer.

### 4.4 Cell isolation

Mononuclear cells were isolated from mouse spleen by gradient centrifugation in Ficoll solution (17‐1440‐02; GE Healthcare). Then splenic B cells were purified by incubation with anti‐CD90.2 mAb (105310; BioLegend) and guinea pig complement (C300‐0500; Rockland) for 30 min. In order to further remove adherent cells, the cells were cultured in the incubator (37°C, 5% CO_2_) for 1 h.

### 4.5 Flow cytometry

For the analysis of splenic B cell subsets, cells were stained with different surface markers. The whole operation was kept on ice and out of light. The antibodies used are as follows: APC‐anti‐CD21 (123412; BioLegend), BV421‐anti‐IgM (406518; BioLegend), BV510‐anti‐B220 (103234; BioLegend), FITC‐anti‐CD19 (101506; BioLegend), PerCP/Cy5.5‐anti‐IgD (405710; BioLegend), FITC‐anti‐CD95 (152606; BioLegend), AF647‐anti‐GL7 (144606; BioLegend), percp‐7AAD (6084701; BD Pharmingen), and PE‐anti‐CD23 (101608; BioLegend). In addition, PE‐Cy7‐anti‐ki67 (1983210; Invitrogen) was used to label cells in the proliferation cycle and Annexin V (640906; BioLegend) was used to detect apoptosis. Data were acquired on Attune NxT (Thermo Fisher) and analyzed by the FlowJo software (TreeStar).

### 4.6 CFm and TIRFm

For confocal analysis, purified splenic B cell suspension was dropped onto the poly‐lysine coated slides and incubated at 37°C for 30 min to make cells adherent. After stimulating cells in a water bath (37°C) with Alexa Fluor 594 F(ab ')2 Goat Anti‐mouse IgM+IgG (H+L) (115586‐068; Jackson ImmunoResearch), cells were fixed with 4% paraformaldehyde at 0 min, 5 min, 10 and 30 min, respectively. Next, cells were permeabilized with 0.05% saponin buffer (S4521‐10G; Sigma–Aldrich), and stained with primary and secondary antibodies successively. All the operations were performed on ice and protected from light. Finally, pictures were taken with Nikon Eclipse Ti‐PFS and analyzed with NIS‐elements AR 5.01 software for MFI and the correlation coefficient of colocalization. Reagents used are listed below: anti‐phosphotyrosine (pY, 05−321; Merck Millipore), anti‐pSHIP1 (3941S; Cell Signaling Technology), anti‐pCD19 (ab63443, Abcam), anti‐pBtk (ab52192; Abcam), anti‐pWASp (A300‐205A; Bethyl Laboratories), ActinGreen 488 ReadyProbes (R37110; Thermo Fisher), Alexa Fluor 405 goat anti‐mouse IgG (A‐31553; Thermo Fisher), Alexa Fluor 488 goat anti‐rabbit IgG (A‐11008; Thermo Fisher), and Alexa Fluor 405 goat anti‐rabbit IgG (A‐31556; Thermo Fisher).

For TIRFm analysis, as described previously,^50^, ^51^ splenic B cells from 33.0 T exposure group and sham group were incubated with Ag‐tethered lipid bilayer and activated in water bath (37°C) for 3, 5, and 7 min, respectively. After fixation and permeabilization, cells were stained with pY, pBtk, pSHIP, F‐actin, and pWASp. Images were taken by TIRFm (Nikon Eclipse Ti‐PFS). For image‐related analysis, MFI and area of contact zone were detected using NIS‐elements AR 5.01 and background fluorescence was subtracted in the measurement of MFI. There were over 50 individual cells analyzed for each set of data.

### 4.7 Western blotting

Isolated splenic B cells from the 33.0 T group and sham group were incubated with Biotin‐Fab’ of anti‐mouse Ig(M+G) on ice for 30 min, followed by continued incubation with streptavidin for 10 min. Cells were then activated at 37°C for the indicated time. After lysing cells with RIPA buffer (P0013B; Beyotime), the cell lysates were separated by SDS‐PAGE and protein bands were loaded onto nitrocellulose membranes through electrophoretic transfer. After blocking the blank sites, membranes were processed with anti‐pCD19 (ab63443; Abcam), anti‐pSHIP (3941S; CST), anti‐pS6 (4856S; CST), anti‐pAkt (4060L; CST), anti‐pFOXO‐1 (9461S; CST), anti‐pWASp (A300‐205A; Bethyl Laboratories), anti‐pMST1 (3681S; CST), anti‐DOCK8 (11622‐1‐AP; Proteintech), and the secondary antibodies were added later. Images were captured from ChemiDoc™XRS+ imaging systems (Bio‐Rad). β‐Actin (60008‐1‐IG‐10; Proteintech) was used as a loading control.

### 4.8 Transcriptome sequencing and data analysis

Transcriptome libraries were constructed using Hieff NGS® Ultima Dual‐mode mRNA Library Prep Kit for Illumina® (Yeasen; 12252ES96) following the manufacturer's instructions. mRNA libraries were sequenced on GenoLab M platform (GeneMind Biosciences LTD.) using a 150‐cycle paired‐end high‐output sequencing mode.

Raw sequencing data (Fastq) were processed through a GeneMind in‐house perl pipeline. After filtering adapter, ploy‐N or low‐quality reads, clean reads were obtained and mapped to the reference genome sequence (mm10/GRCm39.primary) via HISAT2 tools software. Expression quantification and differential expression were performed using StringTie and DESEq2. Pathway enrichment analysis (KEGG) was conducted with the “clusterProfiler” R package.

The RNA‐seq data sets presented in this study can be found in online repository of China National GeneBank DataBase (CNGBdb) with accession number CNP0004667.

### 4.9 Statical analysis

Unpaired two‐tailed Student's *t*‐test was used for comparisons between two groups. GraphPad Prism 8 Software was used to assess statical significance. Experimental values were shown as mean ± standard error of mean (SEM). Statistical significance was set at *p* < 0.05. Statistical significance differences defined as *p < 0.05, **p < 0.01, ***p < 0.001, and ****p < 0.0001.

## AUTHOR CONTRIBUTION

H. G., L. L., and M. X. performed the flow cytometry experiments. H.G. carried out the western blotting. D. K. performed the confocal experiments. Q. C and Y. J. performed the TIRFm experiments. B. Y. established SMF and sham‐exposed mouse models. H. G., X. Z., and J. L. carried out transcriptome sequencing and analysis. H. G. analyzed the data and generated figures. Y. F. drafted the manuscript. C. L., X. Z., G. H., B. Y., F. G., H. F., J. S., L. Y., J. L., and X. D. reviewed and revised the manuscript. X. Z. and C. L. designed and supervised the study. All authors approved the final paper as submitted and agreed to be accountable for all aspects of the work.

## CONFLICT OF INTEREST STATEMENT

Authors Xin Zhang and Juan Lai were employed by GeneMind Biosciences Company Limited, Shenzhen, China. The remaining authors declare that the research was conducted in the absence of any commercial or financial relationships.

## ETHICS STATEMENT

The animal protocols were approved by the ethical and humane committee of Hefei Institutes of Physical Science, Chinese Academy of Sciences and carried out strictly in accordance with the related regulations. (Ethics No. SWYX‐DW‐2021‐22).

## Supporting information

Supporting Information

## Data Availability

The data sets and any other raw data that support the findings of this study are available from the corresponding author upon reasonable request.
