# Supplementary material for: Ultra‐high static magnetic fields cause immunosuppression through disrupting B‐cell peripheral differentiation and negatively regulating BCR signaling
Source: MedComm (2020). 2023 Oct 1;4(5):e379. doi: 10.1002/mco2.379 (PMC10542999; doi:10.1002/mco2.379)
Supplement: Supplementary file 1 — Supporting Information [file MCO2-4-e379-s001.docx]

Ultra-high static magnetic fields cause immunosuppression through disrupting B-cell peripheral differentiation and negatively regulating BCR signaling

Heng Gu^1^, Yufan Fu^1^, Biao Yu^2^, Li Luo^1^, Danqing Kang^1^, Miaomiao Xie^1^, Yukai Jing^1^, Qiuyue Chen^1^, Xin Zhang^3^, Juan Lai^3^, Fei Guan^1^, Huamei Forsman^4^, Junming Shi^1^, Lu Yang^1^, Jiahui Lei^1^, Xingrong Du^5*^, Xin Zhang^2,6*^, and Chaohong Liu^1*^

^1^Department of Pathogen Biology, School of Basic Medicine, Tongji Medical College, Huazhong University of Science and Technology, Wuhan, China.

^2^High Magnetic Field Laboratory, Hefei Institutes of Physical Science, Chinese Academy of Sciences, Hefei, Anhui 230031, China;

^3^GeneMind Biosciences Company Limited, Shenzhen, 518001, China

^4^Department of Rheumatology and Inflammation Research, Institute of Medicine, Sahlgrenska Academy, University of Gothenburg, Sweden.

^5^Shanghai Key Laboratory of Metabolic Remodeling and Health, Institute of Metabolism and Integrative Biology, Fudan University, Shanghai, China

^6^Institutes of Physical Science and Information Technology, Anhui University, Hefei, Anhui, 230601, China

*Corresponding author:

Chaohong Liu

Department of Pathogen Biology, School of Basic Medicine, Tongji Medical College and State Key Laboratory for Diagnosis and Treatment of Severe Zoonostic Infectious Disease, Huazhong University of Science and Technology, Wuhan, 430030, Hubei, China

E-mail: [chaohongliu80@126.com](mailto:chaohongliu80@126.com)

Xin Zhang

High Magnetic Field Laboratory, Hefei Institutes of Physical Science, Chinese Academy of Sciences, Hefei, Anhui 230031, China; Institutes of Physical Science and Information Technology, Anhui University, Hefei, Anhui, 230601, China;

E-mail: [xinzhang@hmfl.ac.cn](mailto:xinzhang@hmfl.ac.cn)

Xingrong Du

Shanghai Key Laboratory of Metabolic Remodeling and Health, Institute of Metabolism and Integrative Biology, Fudan University, Shanghai, China

E-mail: [xingrong_du@fudan.edu.cn](mailto:xingrong_du@fudan.edu.cn)


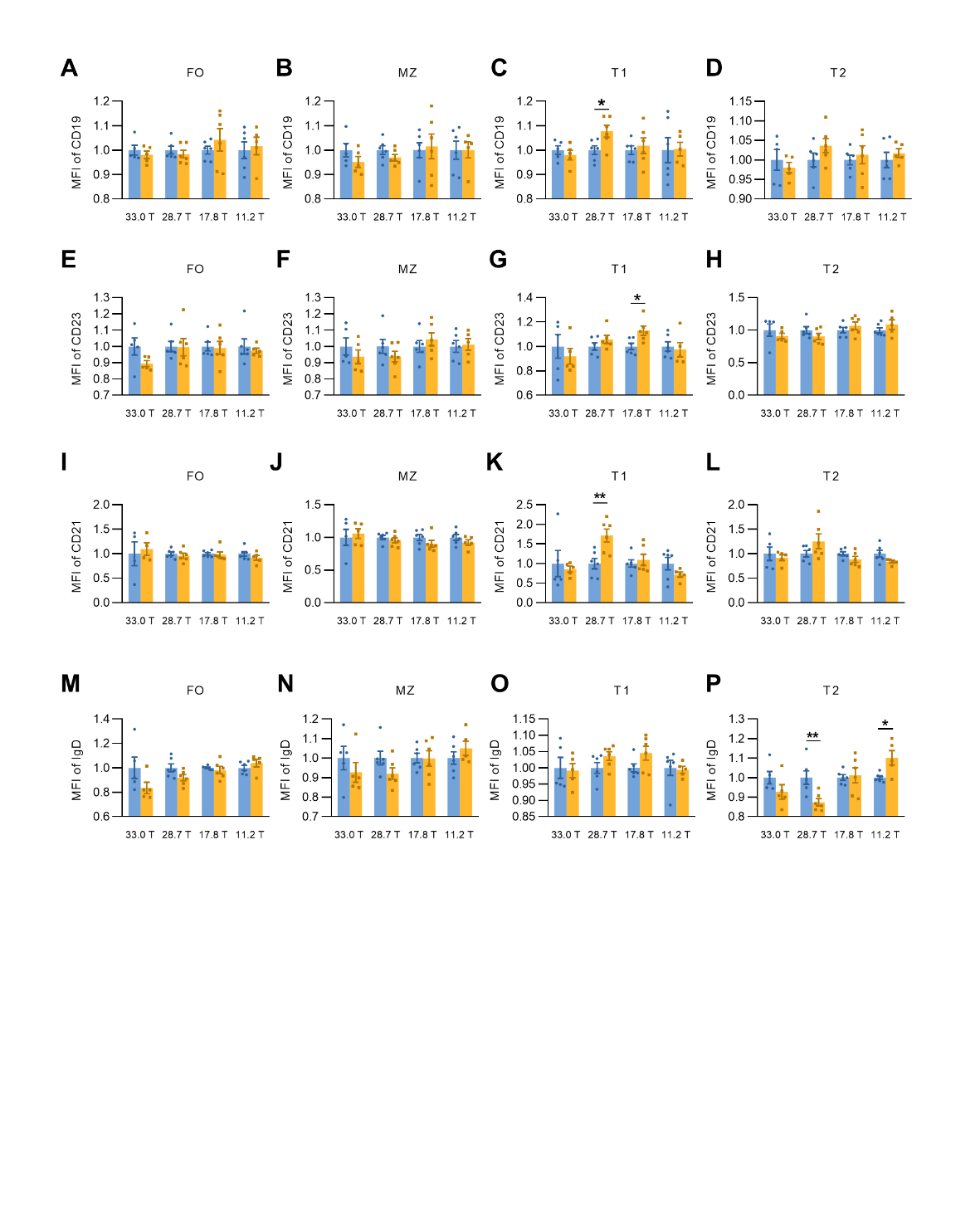


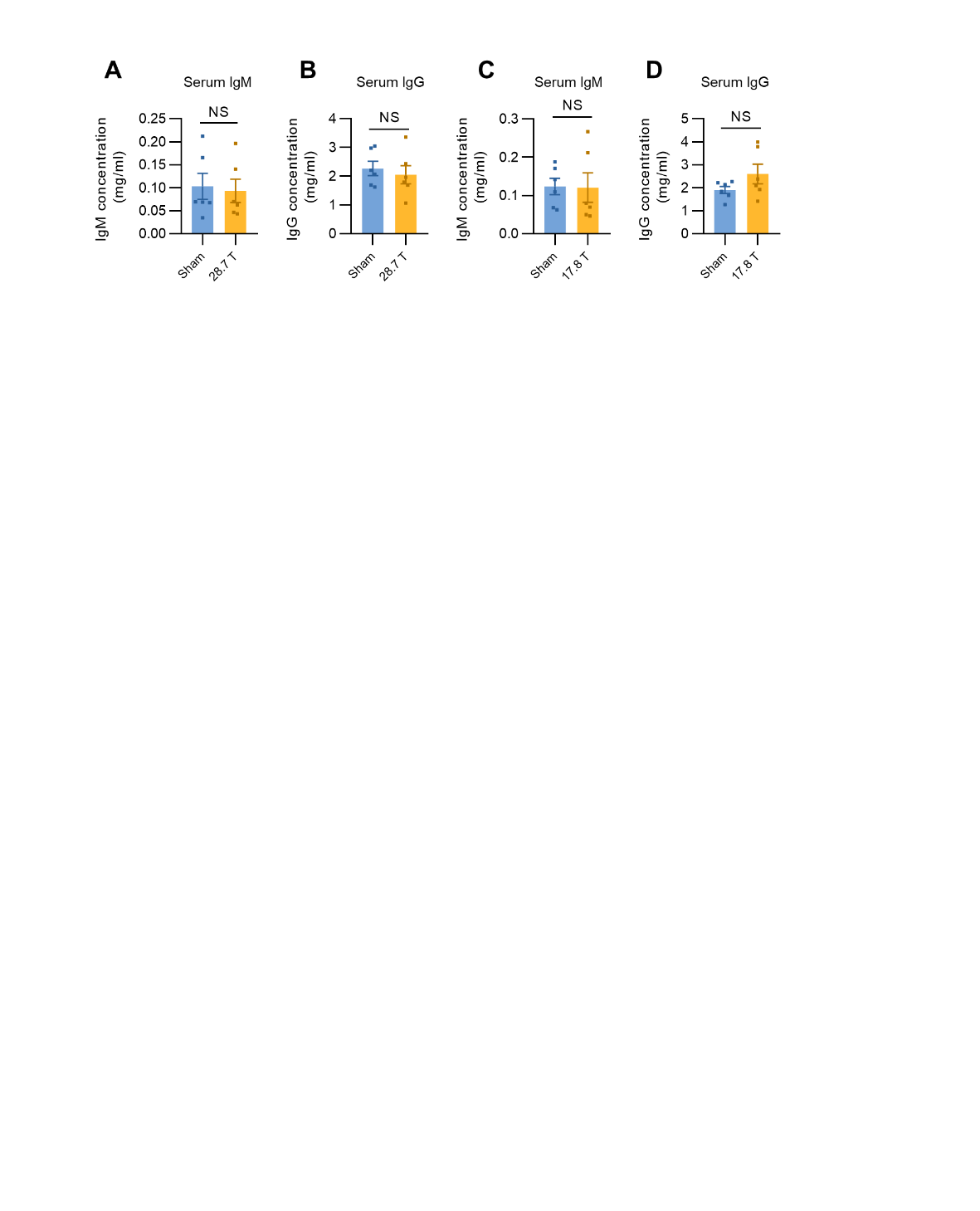


**Figure S1** SMF exposure has almost no effect on the expression of CD19, IgD, CD23 and CD21 on different subset of B cells. **A-D** MFI of CD19 in FO/MZ/T1/T2 B cells from sham and 33.0 T/28.7 T/17.8 T/11.2 T SMF-exposed groups. **E-H** MFI of CD23 in FO/MZ/T1/T2 B cells from sham and 33.0 T/28.7 T/17.8 T/11.2 T SMF-exposed groups. **I-L** MFI of CD21 in FO/MZ/T1/T2 B cells from sham and 33.0 T/28.7 T/17.8 T/11.2 T SMF-exposed groups. **M-P** MFI of IgD in FO/MZ/T1/T2 B cells from sham and 33.0 T/28.7 T/17.8 T/11.2 T SMF-exposed groups. Samples were analyzed with FlowJo software, Error bars were shown as mean ± SEM. Dots represent individual mouse. *P < 0.05; **P < 0.01; ***P < 0.001 and ns: no statistical significance.

**Figure S2** 28.7 T or 17.8 T SMF exposure had little effect on serum IgM/IgG levels in mice. **A-B** The concentration of IgM/IgG in serum of mice in sham and 28.7 T SMF-exposed groups (N = 6). **C-D** The concentration of IgM/IgG in serum of mice in sham and 17.8 T SMF-exposed groups (N = 6). Error bars were shown as mean ± SEM. Dots represent individual mouse. *P < 0.05; **P < 0.01; ***P < 0.001 and ns: no statistical significance.
